# The regulation of methylation on the Z chromosome and the identification of multiple novel Male Hyper-Methylated regions in the chicken

**DOI:** 10.1371/journal.pgen.1010719

**Published:** 2024-03-08

**Authors:** Andrey Höglund, Rie Henriksen, Allison M. Churcher, Carlos M. Guerrero-Bosagna, Alvaro Martinez-Barrio, Martin Johnsson, Per Jensen, Dominic Wright

**Affiliations:** 1 Science for Life Laboratory, Department of Environmental Science, Stockholm University, Stockholm, Sweden; 2 AVIAN Behavioural Genomics and Physiology Group, Linköping University, Linköping, Sweden; 3 NBIS, Umeå University, Department of Molecular Biology, Umeå, Sweden; 4 Physiology and Environmental Toxicology Program, Department of Organismal Biology, Uppsala University, Uppsala, Sweden; 5 NBIS, Uppsala University, SciLifeLab, Uppsala, Sweden; 6 Department of Animal Breeding and Genetics, Swedish University of Agricultural Sciences, Uppsala, Sweden; Netherlands Institute of Ecology, NETHERLANDS

## Abstract

DNA methylation is a key regulator of eukaryote genomes, and is of particular relevance in the regulation of gene expression on the sex chromosomes, with a key role in dosage compensation in mammalian XY systems. In the case of birds, dosage compensation is largely absent, with it being restricted to two small Male Hyper-Methylated (MHM) regions on the Z chromosome. To investigate how variation in DNA methylation is regulated on the Z chromosome we utilised a wild x domestic advanced intercross in the chicken, with both hypothalamic methylomes and transcriptomes assayed in 124 individuals. The relatively large numbers of individuals allowed us to identify additional genomic MHM regions on the Z chromosome that were significantly differentially methylated between the sexes. These regions appear to down-regulate local gene expression in males, but not remove it entirely (unlike the lncRNAs identified in the initial MHM regions). These MHM regions were further tested and the most balanced genes appear to show decreased expression in males, whilst methylation appeared to be far more correlated with gene expression in the less balanced, as compared to the most balanced genes. In addition, quantitative trait loci (QTL) that regulate variation in methylation on the Z chromosome, and those loci that regulate methylation on the autosomes that derive from the Z chromosome were mapped. Trans-effect hotspots were also identified that were based on the autosomes but affected the Z, and also one that was based on the Z chromosome but that affected both autosomal and sex chromosome DNA methylation regulation. We show that both cis and trans loci that originate from the Z chromosome never exhibit an interaction with sex, whereas trans loci originating from the autosomes but affecting the Z chromosome always display such an interaction. Our results highlight how additional MHM regions are actually present on the Z chromosome, and they appear to have smaller-scale effects on gene expression in males. Quantitative variation in methylation is also regulated both from the autosomes to the Z chromosome, and from the Z chromosome to the autosomes.

## Author summary

DNA methylation is a key regulator of eukaryote genomes, and is of particular relevance in the regulation of gene expression on the sex chromosomes, with a key role in dosage compensation in mammalian XY systems. In the case of birds, dosage compensation is largely absent, with it being restricted to two small Male Hyper-Methylated (MHM) regions on the Z chromosome. We utilised a wild x domestic advanced intercross in the chicken, with both hypothalamic methylomes and transcriptomes assayed in 124 individuals, to investigate the role that methylation plays in regulating gene expression on the Z chromosome. Our results highlight how additional MHM regions are actually present on the Z chromosome, and they appear to have smaller-scale effects on gene expression in males. Quantitative variation in methylation is also regulated both from the autosomes to the Z chromosome, and from the Z chromosome to the autosomes. In addition, these MHM regions were further tested and the most balanced genes appear to show decreased expression in males, whilst methylation appeared to be far more correlated with gene expression in the less balanced, as compared to the most balanced genes.

## Introduction

DNA methylation is one of the key regulators of eukaryotic genomes, and can both inhibit [1,2] and enhance gene expression [3,4], depending on where the DNA methylation occurs. This DNA methylation can be environmentally driven [5], but can also be modified and regulated via DNA variation [6,7,8,9,10]. We have previously addressed this using a wild x domestic chicken (*Gallus gallus domesticus*) model to study the regulation of variation in autosomal DNA methylation, and how it can quantitatively regulate gene expression using a QTL mapping based approach. This enabled us to identify how domestication in the chicken led to a small number of large-effect trans hotspots, where these loci regulated variation in DNA methylation throughout the genome. Moreover, we found methylation to not only be the driver but also the response to gene expression variation [4]. However, the corresponding knowledge of regulation of DNA methylation variation on the Z chromosome is still lacking. For example, the extent to which quantitative variation in DNA methylation is controlled between the autosomes and sex chromosomes is an open question, as is the extent to which DNA methylation is regulated on the Z chromosome in general. Given the role that DNA methylation plays in dosage compensation and sex balancing on the Z chromosome in the chicken, this is particularly relevant.

Sex chromosome dosage compensation [11,12] is a process that prevents the expression imbalance originating from the differential number of sex chromosomes present in males or females when homo- and hetero-gametic sexes exist, relative to the autosomes. Dosage compensation occurs when the dose effect due to one sex having only a single sex chromosome, and therefore half the number of gene copies, is compensated by either decreasing gene expression in the homogamete or increasing expression in the heterogamete. This in some ways distinct from sex balancing, which is to balance the expression of genes on the sex chromosomes between males and females. However, the two processes may share at least a partial mechanism in common, in particular in instances where dosage compensation acts to decrease gene expression in the homogametic sex on the sex chromosomes. Although this may harmonise gene expression between the sexes on the sex chromosomes, it also results in half the expression of the sex chromosomes relative to the autosomes. In the case of XX/ XY systems, these have chromosome wide regulation (see [13]), though the mechanism between species is not always the same. In the case of *Drosophila*, up-regulation of the X in males (XY) occurs, resulting in similar expression between males and females for the sex chromosome(s), and also sex chromosomes and autosomes having similar expression, regardless of sex [14]. In the case of placental mammals (also XX/ XY), the X chromosome in the homogametic sex (females) is reduced via X chromosome inactivation [15,16,17]. This results in both sexes having a uniform ratio of expression for the sex chromosomes, and also that autosomes have on average twice the level of expression relative to the sex chromosomes for both sexes (Pessia 2014). This is achieved via epigenetic mechanisms, notably DNA methylation and histone modification [18].

Dosage compensation is less well-described in ZW systems, with the female typically being heterogametic, in contrast to the mammalian XY systems [15,16]. ZZ/ ZW systems lack full global Z chromosome compensation, meaning that the homogametic sex has increased expression of the sex chromosome, relative to the heterogametic sex [19]. Very incomplete and location-specific dosage compensation does appear to occur [20,21,22]. In birds, gene expression on the Z in males (ZZ) is not double that of females, but instead genes on the Z are on average around 30% upregulated in males at the transcript level [20,21,23]. Similarly, males (ZZ), have a similar sex chromosome expression relative to the autosomes, whilst in females this is reduced. In other ZZ/ ZW systems an effect more similar to XX/ XY systems is observed. In the silk moth, *B*. *mori*, the homogametic sex has reduced expression, bringing them in-line with the heterogametic sex chromosome expression, thereby reducing both sexes sex chromosome expression relative to the autosomes [12].

DNA methylation still plays an important role for sex difference regulation on the avian Z chromosome. In particular, the Male Hyper-Methylated (MHM) region at 27.3Mb was first discovered by Teranishi and colleagues [24], whilst more recently an additional region on 73.16–73.17Mb was also identified [25]. With the initial region, it was found that males had greatly increased methylation in an approximately 500kb area, with nine genes that were present there not being expressed in males. In the case of the more recently discovered MHM region at 73.16Mb (designated MHM2), this was smaller and contained three long non coding RNAs (lncRNAs) that were female-biased in expression. In general, these studies are based on small numbers of samples, generally focussing on between species comparisons (for example, one great tit sample was used in Laine et al. [26], two pooled samples from Whole Genome Bisulfite sequenced chicken were used in [27], and one male and one female White Throated Swallow was used in [25]). This makes it harder to detect smaller regions, and in particular the scope of inter-individual variation in these MHM regions. This is concerning, particularly considering the degree of DNA methylation variation across individuals in populations and the role of methylation in phenotype formation [28]. Large-scale analysis of within species variation could give a better resolution of hypermethylated regions as well as detect differences between individuals in sex-specific methylation and gene regulation.

Various questions still remain regarding the MHM regions, and the genes they contain. The sizes of the MHM regions and the effects of the decreased gene expression is particularly noteworthy–are these genes involved in fundamental sex differences? Similarly, are the genes within these MHM regions regulated in a region-by-region basis or on a gene-by-gene basis? Gene expression regulation via methylation is not restricted to solely promoter regions [6], but can affect gene expression (both positively and negatively) due to effects at enhancer sites, Transcriptional Elements (TEs) and the like. For example, our previous study based on autosomal methylation variation in the chicken found that there was a bias to being positively correlated, whilst correlations between methylation and gene expression could be found within a megabase upstream and downstream of the gene itself [4]. Given this, how far away from these MHM regions are genes being affected? Is this still affecting dosage compensation if it upregulates genes?

To investigate how DNA methylation variation is regulated on the Z chromosome, as well as the potential role of methylation in sex-balancing related dosage compensation, we conducted a DNA methylation quantitative trait locus (methylation QTL) analysis using an advanced intercross between domestic chickens and wild Red Junglefowl. We assayed the hypothalamic transcriptome and methylome on the Z chromosome for 124 individuals, having previously assayed the autosomes for these individuals. It was therefore possible to map both *cis* and *trans* related loci that modulate variation in DNA methylation on the Z chromosome, as well as to assess how DNA methylation is used to regulate sex-differences in gene expression on the Z chromosome. In particular, we are interested in how DNA methylation balances the expression of genes on the sex chromosome between males and females (primarily, rather than the balancing of expression between sex chromosomes and autosomes). For this purpose, an intercross between lines that strongly diverge for DNA methylation levels is an excellent model, as it creates a population with a wide degree of variation in both DNA methylation and gene expression. In this case, the origin of the methylation differences (wild vs domestic) is not as important for sex balancing aspects as the wide variation in DNA methylation present, enabling correlations between widely varying DNA methylation and gene expression levels on the Z chromosome to be assessed in both sexes.

We hypothesise that DNA methylation plays a distinct role in sex balancing in the MHM region, beyond its role in dosage compensation. Thus, whilst the MHM region is known for the almost complete suppression of nine lncRNAs in males, it also has the capacity to quantitatively modify Z chromosome gene expression variation. If this is distinct to dosage compensation, then potentially sex balancing can decrease the expression of the homogametic sex. In this case, whilst genes on the Z may be balanced between sexes, they are not compensated between the autosomes and the Z chromosomes in either sex. Further, we hypothesise that the regulation of sex chromosomes by DNA methylation in birds is at least partly regional, as opposed to the global DNA methylation-mediated mechanism described in mammals. One potential benefit of some regional mechanisms is that it allows for the resolution of intra-locus sexual conflict on the sex chromosome, by means of trans-regulation.

The role that DNA methylation plays in sex balancing on the Z chromosome can be investigated by the relative abundance of sex interaction effects in cis and trans methylation QTL that are either present on the Z chromosome (cis), or present on the autosomes but affect DNA methylation on the Z chromosome (trans). Similarly, sex interaction effects in cis and trans expression QTL affecting the Z chromosome can also be investigated. In these cases, sex balancing can be far more easily achieved via sex interaction effects, although these can also interact with dosage compensation effects. We would thus predict that sex interaction effects affecting the Z chromosome should be highly prevalent, though if these effects interact with Z chromosome dosage compensation, then local methylation and expression QTL may possess fewer sex interactions as they are reduced by the compensation effects. Similarly, we would also predict that where sex balancing occurs, this is due to decreased expression of the homogametic sex, rather than increased expression of the heterogametic sex, if sex balancing and dosage compensation are distinct to one another.

## Methods

### Ethics statement

The study was approved by the Local Ethical Committee (Linköping) of the Swedish National Board of Laboratory Animals. Dnr 16110–2020.

### Study population

The study population was composed of 124 chickens (55 females, 69 males) from which the hypothalamus tissue was dissected out at day 212. The individuals used were from an 8th generation advanced intercross, founded using a Red Junglefowl (wild) male and three White Leghorn (domestic) females. A detailed description of the intercross generation, housing conditions, etc can be found in [29].

### RNA and DNA methylation isolation

RNA was isolated from the hypothalamus tissue which was homogenised using Ambion TRI Reagent (Life Technologies) following the manufacturer’s protocol. cDNA synthesis and microarray-based gene expression were performed using a Nimblegen 135k array, as described previously [30]. DNA was isolated from the remainder of the TRI reagent homogenate by mixing 125μl ice-cold 99% ethanol with 250μl TRI reagent homogenate. Samples were vortexed, incubated on ice for 5min and centrifuged at 12’000 RPM for 10 min in room temperature. The pellet was saved and isolation continued using the DNeasy Blood & Tissue Kit (Qiagen) following the manufacturer’s protocol. DNA methylation was assessed by Methylated DNA immunoprecipitation (MeDIP) protocol. Further details of the MeDIP protocol can be found in [4].

### Phenotypes: methylation and gene expression

DNA methylation phenotypes were generated by dividing the chicken genome into 1000bp windows, yielding a total of 1050176 methylation windows, of which 82426 were located on chromosome Z. The MeDIP-seq reads were mapped to each methylation window and normalised by dividing with the total read count for each individual respectively. Sequencing was performed on an IonProton machine (Thermo Fisher Scientific) using the Torrent Suite software (version 4.4.1) by the National Genomics Infrastructure in Uppsala, Sweden. The sequence depth was on average 3.4X ± 0.97 (standard deviation), the read length was on average 136 ± 15 bp, the raw reads was on average 23.8 million ± 5.2 and the quality score was on average 22 ± 1. The Gene expression dataset has been published previously [30] and was based on the NimbleGen 12 x 135K Custom Gene expresson array, mapping to 22628 unique genes composed of Ensembl, RefSeq genes and Expressed Sequence Tags.

### Quantitative Trait Loci (QTL) analysis

Quantitative Trait Loci (QTL) analysis was performed to identify genomic regions associated with the variation found within DNA methylation levels for the 1 million methylation windows. A genetic marker map was generated using 652 SNP markers, of which 542 were fully informative between the original parental animals used to generate the intercross. Average marker distance was ~16 cM, as per recommendations [31]. Of these, 36 markers were present on the Z chromosome with a 15cM average marker distance. Note that as the intercross is a linkage-based cross and not a GWAS of an outbred population (which relies on linkage disequilibrium and has built up historical recombinations over hundreds of generations) far fewer markers are required to cover the genome, as it is only required to identify the recombinations that have accrued during the intercrossing [32]. Details of the genetic marker locations can be found in Johnsson et al [33]. Interval mapping was performed using the “qtl2” R-package [34]. This package was used as it is able to correctly analyse sex chromosomes in an advanced intercross. A local (cis) scan was performed, restricted to methylation windows present on the Z chromosome, with the local region considered to be within 50cM up- and down-stream of each methylation window. A trans scan was also performed. In the case of the trans scan, a full genome scan was performed for trans effect methylation QTL that were located on either the autosomes or Z chromosome that affected methylation on the Z chromosome. In addition, a scan was also performed for trans methylation QTL located on the Z chromosome that were associated with methylation present on the autosomes. Sex and batch were set as covariates in the test model, with sex also used as an interactive covariate, where significant (if the LOD score of the sex interaction model was >1 LOD higher than the non-sex interaction model). Significance thresholds were determined via a permutation test with and without sex interactions for both local (cis) and trans methylation QTL. Local (putatively cis) regions were defined as 50cM up and downstream to the closest genetic marker, whilst anything outside this region was defined as trans. For the trans permutations, 20000 random methylation phenotypes were permuted 1000 times each, both for sex and non-sex interaction, and for cis permutations 17000 random phenotypes permuted 1000 times each. From the permutations the top 5% LOD-scores for each phenotype were saved and from these the top 5% were chosen as significance threshold and the top 20% as the suggestive threshold, respectively. This yielded significance cis LOD-score of 5.73 (sex interaction) and 4.29, (no sex interaction), with suggestive thresholds of 4.87 and 3.58. For the trans thresholds, significance was at LOD-score of 7.70 and 7.68 (sex and non-sex interaction, respectively), whilst the suggestive threshold was 5.92 and 5.93. To prevent QTL that were strongly dependent on outliers, we firstly removed all QTL that had fewer than 10 individuals in any genotype class, and also removed QTL that were driven by large effect single outliers via visual inspection.

Gene expression QTL (eQTL) analysis was performed using R/qtl, using RMA preprocessed [35] expression levels as quantitative phenotypes with sex and batch as additive covariates. The same criteria for cis-eQTL was applied as for the autosomes (see [30], with local eQTL defined as those within +/-50cM of the gene, with trans referring to any other location. Significance thresholds for cis and trans eQTL were 4.0 and 6.0, respectively.

### Male Hyper-Methylated (MHM) region

The MHM region was identified using the transcript deposited in the NCBI GenBank by [24], accession AB046698 (2332 bp), with this being the probe sequence used to identify the region initially. This sequence maps to two genomic locations: chrZ:27375241–27391116 (99.1% match) and chrZ:27329191–27333743 (98.9% match), hereafter referred to as MHMa and MHMb respectively. The MHMa and MHMb regions were corroborated in our dataset and the parameters for methylation levels obtained were used to identify other MHM-like regions. These parameters were: median methylation status per window of > 8.52, a sex difference equal to a Wilcoxon rank sum test/Mann-Whitney test p-value < 1.75e-10 and comprising of five or more adjacent methylation windows (i.e. these values are those identified for the original MHM region in our dataset).

### QTL overlaps

With both mQTL and eQTL mapped it was possible to assess whether any correlations could be found between DNA methylation levels and gene expression which are both associated to a locus. By overlapping the confidence intervals of the mQTL and eQTL, and regressing the gene expression with methylation, genomic regions that putatively control either the methylation or gene expression (or both) were observed. The correlation was tested with all individuals and sex as a factor, and with the sexes separate, yielding 3 models. Any genes that significantly correlated with a methylation window were finally tested for causality using the Network Edge Orientation (NEO) package in R [36]. In this way, it is possible to ascribe hypothetical orientation of the regulatory relationship, whether DNA methylation regulates gene expression or vice versa. Significance using the NEO package is based on the LEO.NB score, which quantifies the support of the best fitting causal model versus the second best fitting model. NEO assesses causality by using the SNP markers from each QTL as anchors that are then used for orientating the edges of the network and integrating gene expression and DNA methylation, through the use of Structural Equation Model comparisons (SEM). The extent of edge orientation is calculated with a Local SEM-based Edge Orienting score (LEO), that is compared to the next best model (NB). As both the eQTL and methylation QTL originated from the same genotype (imputed marker position) and thus are treated as a single-marker orientation with a LEO.NB.OCA-score > 1.0 considered significant, and a score of > 0.8 as suggestive.

Direction of causality was tested as follows:

1. eQTL marker -> gene expression -> DNA methylation <- methQTL marker
2. eQTL marker -> gene expression <- DNA methylation <- methQTL marker
3. eQTL marker -> gene expression -> DNA methylation
4. gene expression <- DNA methylation <- methQTL marker
5. gene expression <- shared QTL marker -> DNA methylation
6. gene expression -> DNA methylation <- methQTL marker
7. eQTL marker -> gene expression <- DNA methylation

## Results

### Dosage Compensation and the Male Hyper Methylated (MHM) Region

**Fig 1:**
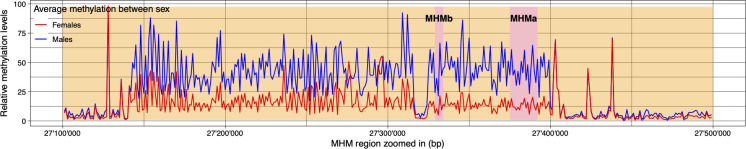
MHMa and MHMb regions (used to identify the original MHM region) and sex differences in methylation levels at these and the surrounding area. Male methylation is shown in blue, female methylation is shown in red.

To assess the degree of male-biased hyper methylated regions, we first analysed the previously known hyper methylated regions–MHMa and MHMb. These two regions, situated at 27.375Mb and 27.329Mb respectively, had a 3.3 and 3.6-fold increase in methylation in males, respectively, with these ratios being highly significant (max Wilcoxon pvalue < 4e-10 and 1.7e-10, respectively, for each region), see S1 Table). The original MHM region was hypothesised to be approximately 460kb in length [24]. When we assessed the methylation around these two regions, we find elevated methylation from 27.142Mb-27.40Mb (259-kb long), more accurately demarking this region, see Fig 1. To identify further male biased methylation windows, we performed a chromosome-wide scan calculating the degree of sex bias. Based on the pre-existing MHM region, we then selected all those regions with both a strongly significant sex bias (p<1.75e-10, as compared to the average sex bias in methylation on the Z chromosome being p = 0.14 and a 1.69 fold methylation difference between males and females) and with at least five adjacent methylation windows (see methods section).

**Fig 2:**
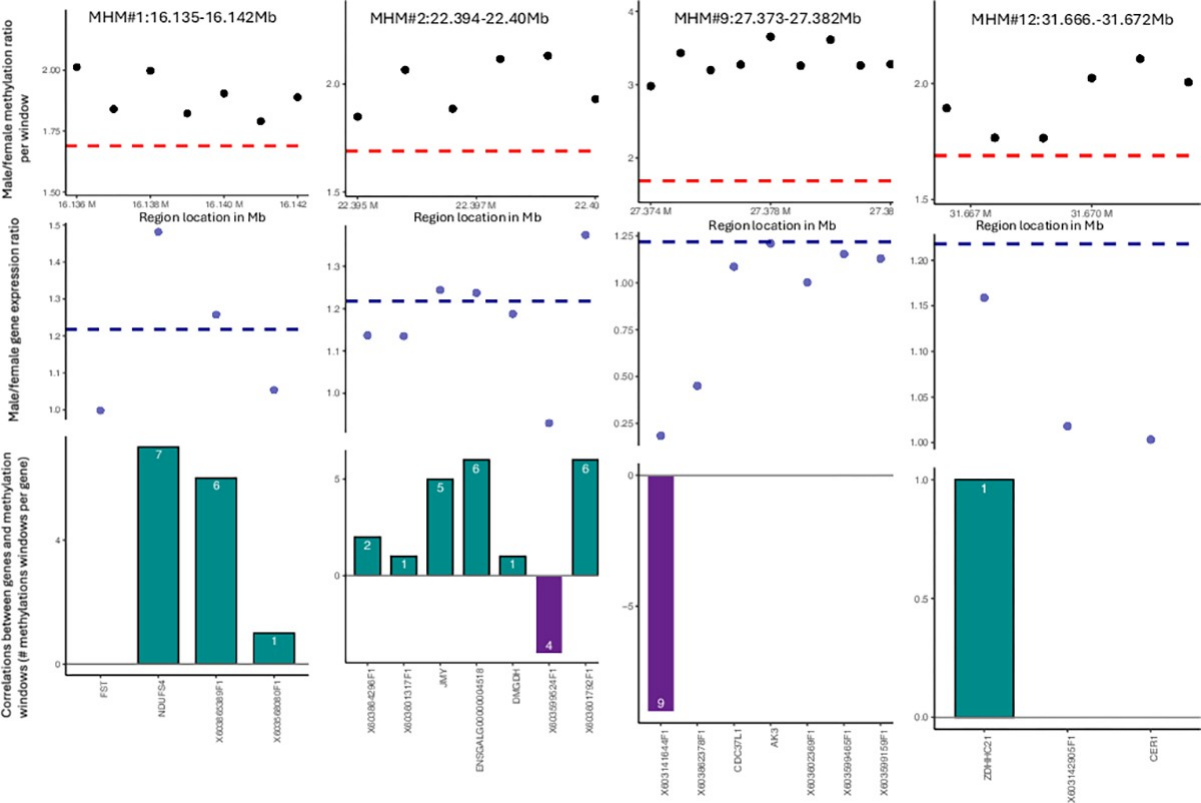
Four of the 19 Novel MHM regions present on the Z chromosome and their effects on gene expression. Panes illustrate regions 1, 2, 9, 12 (selected as being representative of all the regions). Each pane consists of the following: i) The male:female methylation ratio for the 1kb methylation windows that make up the MHM region (each black dot represents the ratio at one methylation window). The red hashed line at the base indicates the average male:female methylation ratio (~1.7). ii) Male:female gene expression ratio is indicated by the blue dots, one for each gene in the region, with the ratio shown on the left-side y-axis, and the blue hashed line indicating the average male:female gene expression ratio on the Z chromosome (~1.2). iii) The number of correlations between each gene and the 1kb methylation windows that make up each MHM. The direction of the correlation (positive or negative) is indicated by the bar being above the line (positive, coloured turquoise) or below the line (negative, coloured purple). The number of correlations is indicated on each bar, whilst each gene name is given on the x-axis.

In total, 19 MHM regions (hereafter referred to as blocks) were identified (see Table 1 and Figs 2 and S1). Of these continuous blocks, 17 had genes in the local vicinity. In this instance, we defined local as being within 100kb of the MHM block, as in our previous study we found strong correlations between gene expression and DNA methylation even up to 100kb away from the gene itself. To test if dosage compensation acts locally on a gene-by-gene basis or uniformly throughout each block, the methylation levels within these MHM blocks were correlated with the neighbouring genes (see Table 1**),** i.e. individual methylation windows present within each block were correlated with the expression of adjacent genes, controlling for multiple testing. Of the 17 blocks with adjacent genes, 14 had a significant correlation between at least one methylation window and local gene expression, see Figs 2 and S1. Interestingly, neighbouring genes frequently displayed different correlations with methylation (i.e. neighbouring genes could have very different correlations with local DNA methylation), indicating that these regions seem to be associated with expression on a gene-by-gene basis.

**Table 1:** Novel Male Hyper-Methylated (MHM) regions identified in the hypothalamus. The 17 MHM regions containing genes are divided into separate regions, with their location, size, number of probesets present initially given. Also included are the average gene expression values for males and females, the p-value of the sex differences in gene expression, the ratio of male:female gene expression, the number of 1kb windows present within the MHM region that correlate with each gene and the direction of that correlation.

MHM Regions			Gene Expression											meth ~ genexp corr		
MHM #	pos	size (bp)	chr	start	end	ensembl/ probeset	name	info	avg exp.	avg exp. male	avg exp. female	wilcox pval	M:F ratio	# corr	# neg. Tval	# pos. Tval
1	chrZ:16135000–16142000	7000	Z	1.6E+07	1.6E+07	ENSGALG00000014908	FST	follistatin [Source:NCBI gene;Acc:396119]	342.26	341.96	342.64	1.00E+00	1	0	0	0
1	chrZ:16135000–16142000	7000	Z	1.6E+07	1.6E+07	ENSGALG00000028026	NDUFS4	NADH:ubiquinone oxidoreductase subunit S4 [Source:HGNC Symbol;Acc:HGNC:7711]	3515.57	4107.78	2772.6	7.50E-07	1.48	7	0	7
1	chrZ:16135000–16142000	7000	Z	1.6E+07	1.6E+07	ENSGALG00000053142			#N/A	#N/A	#N/A	#N/A	#N/A	0	0	0
1	chrZ:16135000–16142000	7000	Z	1.6E+07	1.6E+07	#N/A	#N/A	#N/A	159.84	175.81	139.79	6.30E-02	1.26	6	0	6
1	chrZ:16135000–16142000	7000	Z	1.6E+07	1.6E+07	#N/A	#N/A	#N/A	232.83	238.2	226.09	1.00E+00	1.05	0	0	0
2	chrZ:22394000–22400000	6000	Z	2.2E+07	2.2E+07	#N/A	#N/A	#N/A	153.42	162.07	142.57	1.00E+00	1.14	2	0	2
2	chrZ:22394000–22400000	6000	Z	2.2E+07	2.2E+07	#N/A	#N/A	#N/A	2818.28	2975.23	2621.37	1.30E-03	1.13	2	0	2
2	chrZ:22394000–22400000	6000	Z	2.2E+07	2.2E+07	ENSGALG00000014819	JMY	junction mediating and regulatory protein, p53 cofactor [Source:HGNC Symbol;Acc:HGNC:28916]	633.88	694.36	558	5.20E-02	1.24	6	0	6
2	chrZ:22394000–22400000	6000	Z	2.2E+07	2.2E+07	ENSGALG00000050556		translation initiation factor IF-2-like [Source:NCBI gene;Acc:101748033]	#N/A	#N/A	#N/A	#N/A	#N/A	0	0	0
2	chrZ:22394000–22400000	6000	Z	2.2E+07	2.2E+07	ENSGALG00000004518		betaine—homocysteine S-methyltransferase 2 [Source:NCBI gene;Acc:416371]	3902.54	4265.37	3447.35	1.70E-04	1.24	6	0	6
2	chrZ:22394000–22400000	6000	Z	2.2E+07	2.2E+07	ENSGALG00000004491	DMGDH	dimethylglycine dehydrogenase [Source:HGNC Symbol;Acc:HGNC:24475]	761.17	818.52	689.21	4.90E-01	1.19	2	0	2
2	chrZ:22394000–22400000	6000	Z	2.2E+07	2.2E+07	#N/A	#N/A	#N/A	38.39	37.1	39.93	1.00E+00	0.93	4	4	0
2	chrZ:22394000–22400000	6000	Z	2.2E+07	2.2E+07	#N/A	#N/A	#N/A	212.49	241.69	175.84	3.10E-03	1.37	6	0	6
3	chrZ:27141000–27149000	8000	Z	2.7E+07	2.7E+07	#N/A	#N/A	#N/A	101	104.55	96.61	1.00E+00	1.08	0	0	0
3	chrZ:27141000–27149000	8000	Z	2.7E+07	2.7E+07	ENSGALG00000010187	SLC1A1	solute carrier family 1 member 1 [Source:HGNC Symbol;Acc:HGNC:10939]	556.46	540.19	576.88	1.00E+00	0.94	0	0	0
3	chrZ:27141000–27149000	8000	Z	2.7E+07	2.7E+07	#N/A	#N/A	#N/A	368.04	364.47	372.52	1.00E+00	0.98	0	0	0
3	chrZ:27141000–27149000	8000	Z	2.7E+07	2.7E+07	#N/A	#N/A	#N/A	28.85	28.79	28.92	1.00E+00	1	0	0	0
3	chrZ:27141000–27149000	8000	Z	2.7E+07	2.7E+07	ENSGALG00000053562		uncharacterized LOC112530632 [Source:NCBI gene;Acc:112530632]	#N/A	#N/A	#N/A	#N/A	#N/A	0	0	0
4	chrZ:27150000–27160000	10000	Z	2.7E+07	2.7E+07	#N/A	#N/A	#N/A	101	104.55	96.61	1.00E+00	1.08	0	0	0
4	chrZ:27150000–27160000	10000	Z	2.7E+07	2.7E+07	ENSGALG00000010187	SLC1A1	solute carrier family 1 member 1 [Source:HGNC Symbol;Acc:HGNC:10939]	556.46	540.19	576.88	1.00E+00	0.94	0	0	0
4	chrZ:27150000–27160000	10000	Z	2.7E+07	2.7E+07	#N/A	#N/A	#N/A	368.04	364.47	372.52	1.00E+00	0.98	0	0	0
4	chrZ:27150000–27160000	10000	Z	2.7E+07	2.7E+07	#N/A	#N/A	#N/A	28.85	28.79	28.92	1.00E+00	1	0	0	0
4	chrZ:27150000–27160000	10000	Z	2.7E+07	2.7E+07	ENSGALG00000053562		uncharacterized LOC112530632 [Source:NCBI gene;Acc:112530632]	#N/A	#N/A	#N/A	#N/A	#N/A	0	0	0
4	chrZ:27150000–27160000	10000	Z	2.7E+07	2.7E+07	ENSGALG00000048068			#N/A	#N/A	#N/A	#N/A	#N/A	0	0	0
5	chrZ:27236000–27245000	9000	Z	2.7E+07	2.7E+07	ENSGALG00000053562		uncharacterized LOC112530632 [Source:NCBI gene;Acc:112530632]	#N/A	#N/A	#N/A	#N/A	#N/A	0	0	0
5	chrZ:27236000–27245000	9000	Z	2.7E+07	2.7E+07	ENSGALG00000050012			#N/A	#N/A	#N/A	#N/A	#N/A	0	0	0
5	chrZ:27236000–27245000	9000	Z	2.7E+07	2.7E+07	ENSGALG00000048068			#N/A	#N/A	#N/A	#N/A	#N/A	0	0	0
5	chrZ:27236000–27245000	9000	Z	2.7E+07	2.7E+07	#N/A	#N/A	#N/A	1660.78	559.76	3042.07	1.40E-07	0.18	9	9	0
5	chrZ:27236000–27245000	9000	Z	2.7E+07	2.7E+07	#N/A	#N/A	#N/A	170.5	110.64	245.6	4.60E-09	0.45	9	9	0
6	chrZ:27257000–27267000	10000	Z	2.7E+07	2.7E+07	ENSGALG00000050012			#N/A	#N/A	#N/A	#N/A	#N/A	0	0	0
6	chrZ:27257000–27267000	10000	Z	2.7E+07	2.7E+07	ENSGALG00000048068			#N/A	#N/A	#N/A	#N/A	#N/A	0	0	0
6	chrZ:27257000–27267000	10000	Z	2.7E+07	2.7E+07	#N/A	#N/A	#N/A	1660.78	559.76	3042.07	1.40E-07	0.18	10	10	0
6	chrZ:27257000–27267000	10000	Z	2.7E+07	2.7E+07	#N/A	#N/A	#N/A	170.5	110.64	245.6	4.60E-09	0.45	10	10	0
7	chrZ:27324000–27350000	26000	Z	2.7E+07	2.7E+07	ENSGALG00000048068			#N/A	#N/A	#N/A	#N/A	#N/A	0	0	0
7	chrZ:27324000–27350000	26000	Z	2.7E+07	2.7E+07	ENSGALG00000051419			#N/A	#N/A	#N/A	#N/A	#N/A	0	0	0
7	chrZ:27324000–27350000	26000	Z	2.7E+07	2.7E+07	#N/A	#N/A	#N/A	1660.78	559.76	3042.07	1.40E-07	0.18	26	26	0
7	chrZ:27324000–27350000	26000	Z	2.7E+07	2.7E+07	#N/A	#N/A	#N/A	170.5	110.64	245.6	4.60E-09	0.45	26	26	0
8	chrZ:27351000–27368000	17000	Z	2.7E+07	2.7E+07	ENSGALG00000051419			#N/A	#N/A	#N/A	#N/A	#N/A	0	0	0
8	chrZ:27351000–27368000	17000	Z	2.7E+07	2.7E+07	#N/A	#N/A	#N/A	1660.78	559.76	3042.07	1.40E-07	0.18	17	17	0
8	chrZ:27351000–27368000	17000	Z	2.7E+07	2.7E+07	#N/A	#N/A	#N/A	170.5	110.64	245.6	4.60E-09	0.45	16	16	0
8	chrZ:27351000–27368000	17000	Z	2.7E+07	2.7E+07	ENSGALG00000014448	CDC37L1	cell division cycle 37 like 1 [Source:HGNC Symbol;Acc:HGNC:17179]	581.96	603.15	555.38	3.90E-01	1.09	0	0	0
8	chrZ:27351000–27368000	17000	Z	2.7E+07	2.7E+07	#N/A	#N/A	#N/A	31.57	31.6	31.54	1.00E+00	1	0	0	0
9	chrZ:27373000–27382000	9000	Z	2.7E+07	2.7E+07	ENSGALG00000051419			#N/A	#N/A	#N/A	#N/A	#N/A	0	0	0
9	chrZ:27373000–27382000	9000	Z	2.7E+07	2.7E+07	#N/A	#N/A	#N/A	1660.78	559.76	3042.07	1.40E-07	0.18	9	9	0
9	chrZ:27373000–27382000	9000	Z	2.7E+07	2.7E+07	#N/A	#N/A	#N/A	170.5	110.64	245.6	4.60E-09	0.45	5	5	0
9	chrZ:27373000–27382000	9000	Z	2.7E+07	2.7E+07	ENSGALG00000014448	CDC37L1	cell division cycle 37 like 1 [Source:HGNC Symbol;Acc:HGNC:17179]	581.96	603.15	555.38	3.90E-01	1.09	0	0	0
9	chrZ:27373000–27382000	9000	Z	2.7E+07	2.7E+07	ENSGALG00000014447	AK3	adenylate kinase 3 [Source:HGNC Symbol;Acc:HGNC:17376]	2604.12	2819.37	2334.08	2.00E-02	1.21	0	0	0
9	chrZ:27373000–27382000	9000	Z	2.7E+07	2.7E+07	#N/A	#N/A	#N/A	31.57	31.6	31.54	1.00E+00	1	0	0	0
9	chrZ:27373000–27382000	9000	Z	2.7E+07	2.7E+07	#N/A	#N/A	#N/A	3094.4	3287.64	2851.97	1.10E-03	1.15	0	0	0
9	chrZ:27373000–27382000	9000	Z	2.7E+07	2.7E+07	#N/A	#N/A	#N/A	263.39	277.56	245.86	9.80E-01	1.13	0	0	0
10	chrZ:27385000–27392000	7000	Z	2.7E+07	2.7E+07	ENSGALG00000051419			#N/A	#N/A	#N/A	#N/A	#N/A	0	0	0
10	chrZ:27385000–27392000	7000	Z	2.7E+07	2.7E+07	#N/A	#N/A	#N/A	1660.78	559.76	3042.07	1.40E-07	0.18	7	7	0
10	chrZ:27385000–27392000	7000	Z	2.7E+07	2.7E+07	#N/A	#N/A	#N/A	170.5	110.64	245.6	4.60E-09	0.45	0	0	0
10	chrZ:27385000–27392000	7000	Z	2.7E+07	2.7E+07	ENSGALG00000014448	CDC37L1	cell division cycle 37 like 1 [Source:HGNC Symbol;Acc:HGNC:17179]	581.96	603.15	555.38	3.90E-01	1.09	0	0	0
10	chrZ:27385000–27392000	7000	Z	2.7E+07	2.7E+07	ENSGALG00000014447	AK3	adenylate kinase 3 [Source:HGNC Symbol;Acc:HGNC:17376]	2604.12	2819.37	2334.08	2.00E-02	1.21	0	0	0
10	chrZ:27385000–27392000	7000	Z	2.7E+07	2.7E+07	#N/A	#N/A	#N/A	31.57	31.6	31.54	1.00E+00	1	0	0	0
10	chrZ:27385000–27392000	7000	Z	2.7E+07	2.7E+07	#N/A	#N/A	#N/A	3094.4	3287.64	2851.97	1.10E-03	1.15	0	0	0
10	chrZ:27385000–27392000	7000	Z	2.7E+07	2.7E+07	#N/A	#N/A	#N/A	263.39	277.56	245.86	9.80E-01	1.13	0	0	0
11	chrZ:27393000–27399000	6000	Z	2.7E+07	2.7E+07	ENSGALG00000051419			#N/A	#N/A	#N/A	#N/A	#N/A	0	0	0
11	chrZ:27393000–27399000	6000	Z	2.7E+07	2.7E+07	#N/A	#N/A	#N/A	1660.78	559.76	3042.07	1.40E-07	0.18	6	6	0
11	chrZ:27393000–27399000	6000	Z	2.7E+07	2.7E+07	#N/A	#N/A	#N/A	170.5	110.64	245.6	4.60E-09	0.45	5	5	0
11	chrZ:27393000–27399000	6000	Z	2.7E+07	2.7E+07	ENSGALG00000014448	CDC37L1	cell division cycle 37 like 1 [Source:HGNC Symbol;Acc:HGNC:17179]	581.96	603.15	555.38	3.90E-01	1.09	0	0	0
11	chrZ:27393000–27399000	6000	Z	2.7E+07	2.7E+07	ENSGALG00000014447	AK3	adenylate kinase 3 [Source:HGNC Symbol;Acc:HGNC:17376]	2604.12	2819.37	2334.08	2.00E-02	1.21	0	0	0
11	chrZ:27393000–27399000	6000	Z	2.7E+07	2.7E+07	#N/A	#N/A	#N/A	31.57	31.6	31.54	1.00E+00	1	0	0	0
11	chrZ:27393000–27399000	6000	Z	2.7E+07	2.7E+07	#N/A	#N/A	#N/A	3094.4	3287.64	2851.97	1.10E-03	1.15	0	0	0
11	chrZ:27393000–27399000	6000	Z	2.7E+07	2.7E+07	#N/A	#N/A	#N/A	263.39	277.56	245.86	9.80E-01	1.13	0	0	0
11	chrZ:27393000–27399000	6000	Z	2.7E+07	2.7E+07	ENSGALG00000053602			#N/A	#N/A	#N/A	#N/A	#N/A	0	0	0
12	chrZ:31666000–31672000	6000	Z	3.2E+07	3.2E+07	ENSGALG00000054940			#N/A	#N/A	#N/A	#N/A	#N/A	0	0	0
12	chrZ:31666000–31672000	6000	Z	3.2E+07	3.2E+07	ENSGALG00000027428	ZDHHC21	zinc finger DHHC-type containing 21 [Source:NCBI gene;Acc:427356]	600.52	639.38	551.76	3.30E-01	1.16	1	0	1
12	chrZ:31666000–31672000	6000	Z	3.2E+07	3.2E+07	#N/A	#N/A	#N/A	34.7	34.97	34.35	1.00E+00	1.02	0	0	0
12	chrZ:31666000–31672000	6000	Z	3.2E+07	3.2E+07	ENSGALG00000049291			#N/A	#N/A	#N/A	#N/A	#N/A	0	0	0
12	chrZ:31666000–31672000	6000	Z	3.2E+07	3.2E+07	ENSGALG00000005434	CER1	cerberus 1, DAN family BMP antagonist [Source:NCBI gene;Acc:395623]	74.21	74.32	74.07	1.00E+00	1	0	0	0
13	chrZ:35555000–35561000	6000	Z	3.6E+07	3.6E+07	ENSGALG00000015138	ABHD17B	transmembrane protein 2 [Source:NCBI gene;Acc:427250]	1098.95	1208.65	961.34	1.10E-04	1.26	6	0	6
13	chrZ:35555000–35561000	6000	Z	3.6E+07	3.6E+07	ENSGALG00000026001	C9orf85	chromosome 9 open reading frame 85 [Source:HGNC Symbol;Acc:HGNC:28784]	850.04	965.8	704.82	8.20E-03	1.37	5	0	5
14	chrZ:38550000–38557000	7000	Z	3.8E+07	3.8E+07	ENSGALG00000052137			#N/A	#N/A	#N/A	#N/A	#N/A	0	0	0
14	chrZ:38550000–38557000	7000	Z	3.8E+07	3.8E+07	#N/A	#N/A	#N/A	182.51	202	158.05	1.50E-02	1.28	7	0	7
14	chrZ:38550000–38557000	7000	Z	3.8E+07	3.9E+07	ENSGALG00000015184	TLE4	transducin like enhancer of split 4 [Source:NCBI gene;Acc:374080]	1149.41	1253.05	1019.39	9.30E-01	1.23	0	0	0
14	chrZ:38550000–38557000	7000	Z	3.9E+07	3.9E+07	#N/A	#N/A	#N/A	656.46	668.55	641.3	1.00E+00	1.04	0	0	0
14	chrZ:38550000–38557000	7000	Z	3.9E+07	3.9E+07	#N/A	#N/A	#N/A	111.28	118.01	102.83	1.00E+00	1.15	0	0	0
15	chrZ:52025000–52031000	6000	Z	5.2E+07	5.2E+07	ENSGALG00000015311	RNF38	ring finger protein 38 [Source:HGNC Symbol;Acc:HGNC:18052]	5893.88	6385.04	5277.7	1.30E-03	1.21	3	0	3
15	chrZ:52025000–52031000	6000	Z	5.2E+07	5.2E+07	ENSGALG00000015315	TRIM14	tripartite motif containing 14 [Source:NCBI gene;Acc:427282]	247.75	265.69	225.9	3.50E-01	1.18	0	0	0
15	chrZ:52025000–52031000	6000	Z	5.2E+07	5.2E+07	ENSGALG00000015320	NANS	N-acetylneuraminate synthase [Source:NCBI gene;Acc:427283]	3893.37	4087.64	3649.65	1.00E-03	1.12	5	0	5
16	chrZ:52073000–52079000	6000	Z	5.2E+07	5.2E+07	ENSGALG00000015311	RNF38	ring finger protein 38 [Source:HGNC Symbol;Acc:HGNC:18052]	5893.88	6385.04	5277.7	1.30E-03	1.21	5	0	5
16	chrZ:52073000–52079000	6000	Z	5.2E+07	5.2E+07	ENSGALG00000015315	TRIM14	tripartite motif containing 14 [Source:NCBI gene;Acc:427282]	247.75	265.69	225.9	3.50E-01	1.18	0	0	0
16	chrZ:52073000–52079000	6000	Z	5.2E+07	5.2E+07	ENSGALG00000015320	NANS	N-acetylneuraminate synthase [Source:NCBI gene;Acc:427283]	3893.37	4087.64	3649.65	1.00E-03	1.12	6	0	6
16	chrZ:52073000–52079000	6000	Z	5.2E+07	5.2E+07	ENSGALG00000015326	CLTA	clathrin light chain A [Source:NCBI gene;Acc:427284]	23451.7	25526.3	20849.1	2.30E-07	1.22	6	0	6
16	chrZ:52073000–52079000	6000	Z	5.2E+07	5.2E+07	#N/A	#N/A	#N/A	24880.1	27004.9	22214.3	1.80E-07	1.22	6	0	6
16	chrZ:52073000–52079000	6000	Z	5.2E+07	5.2E+07	#N/A	#N/A	#N/A	199.25	244.38	142.62	1.90E-03	1.71	5	0	5
19	chrZ:58494000–58500000	6000	Z	5.9E+07	5.9E+07	ENSGALG00000050984		uncharacterized LOC112530550 [Source:NCBI gene;Acc:112530550]	#N/A	#N/A	#N/A	#N/A	#N/A	0	0	0
19	chrZ:58494000–58500000	6000	Z	5.9E+07	5.9E+07	#N/A	#N/A	#N/A	74.27	75.22	73.09	1.00E+00	1.03	0	0	0
19	chrZ:58494000–58500000	6000	Z	5.9E+07	5.9E+07	ENSGALG00000027907	NR2F1	nuclear receptor subfamily 2 group F member 1 [Source:NCBI gene;Acc:100859519]	#N/A	#N/A	#N/A	#N/A	#N/A	0	0	0
19	chrZ:58494000–58500000	6000	Z	5.9E+07	5.9E+07	ENSGALG00000050798			#N/A	#N/A	#N/A	#N/A	#N/A	0	0	0

In total, 51 unique genes (38 present in our dataset) were adjacent to these MHM-like blocks, with 224 significant correlations with methylation levels (methylation windows) of which 134 correlations were negative and 90 positive (tvalue from linear model). Furthermore, of the 38 genes present in our dataset, 34 had a significant sex bias expression with 20 being expressed higher in males and 14 higher in females (M:F ratio). The average fold difference between males and females on the Z chromosome was 1.22 while for the autosomes this was 1.02. In the case of the original MHM region, apart from the RNAse genes (EST probes X603141644 and X603862378 for the lncRNA *ENSGALG00000051419* in Fig 2) that are almost entirely silent in males, this region (see Figs 2 and S1 and Table 1) also contains multiple genes that are still male-biased, but below the average degree of male-bias on the Z chromosome. Similarly, these genes tend to be positively correlated with local methylation, where such a correlation exists. This pattern is also replicated in the newly identified MHM regions (see MHM#1 and #2 in Fig 2, and MHM#12,13,14,15,16,19 in S1 Fig). Therefore, increased methylation in males is associated with a reduction in the differences in male-biased gene expression, but does not eliminate it entirely, in both the existing and the new MHM regions. None of the methylation QTL detected on the Z chromosome (either QTL or phenotypes) overlapped with these MHM regions.

One other MHM region has previously been putatively identified at 73.16–73.173Mb on the Z chromosome by Sun et al. [25]. We also identify this region in our data, though the median methylation threshold fell slightly below the threshold we set, and was therefore excluded initially (i.e. there was a strongly significant sex-difference, but the median level of methylation over all individuals was lower than in the original MHM region). Nevertheless, the region shows very significant DNA methylation levels differences between the sexes (see S2 Table), with significantly more male DNA methylation. All of the neighbouring genes to these MHM regions were also assessed for potential GO enrichment, with no GO enrichment found for those genes in the immediate vicinity.

### Female Hyper-Methylated Regions

As well as additional MHM regions, a search for regions with a lower than average male: female methylation ratio was also performed to identify regions that showed a relative decrease in DNA methylation in males or an increase in DNA methylation in females. Using a criterion of a significant increase in female methylation, relative to males, we firstly identified a total of 118 1kb windows that were significantly more methylated in females than males (see Tables 2 and S3). Of these, three regions consisted of five or more consecutive female-biased methylated windows. These regions were located at 30195000-30200000bp, 42633000-42638000bp, and 49073000-49073000bp on the Z chromosome. No genes were found in these regions, however. An overlap between methylation QTL and these regions was also performed, though once again no overlaps occurred.

**Table 2:** Novel Female Hyper-Methylated regions identified in the hypothalamus. The position, average and median methylation per window, and the average methylation in males and females per window are all given, as well as the significance of the sex-difference and the average male:female fold ratio.

methbin	chr	pos	avg	median	avg_male	avg_female	pvalue	MF_avg	MF_runavg	FHM_blockid
chrZ_30195000	Z	30195000	80.17	70.52	71.05	91.62	1.41E-02	0.78	1.26	1
chrZ_30196000	Z	30196000	52.73	48.01	42.69	65.32	2.07E-05	0.65	1.25	1
chrZ_30197000	Z	30197000	78.65	73.65	70.17	89.28	7.32E-03	0.79	1.23	1
chrZ_30198000	Z	30198000	66.48	60.78	56.40	79.13	2.62E-04	0.71	1.20	1
chrZ_30199000	Z	30199000	69.29	61.94	61.36	79.25	1.64E-02	0.77	1.18	1
chrZ_30200000	Z	30200000	32.70	30.17	28.75	37.65	1.92E-02	0.76	1.16	1
chrZ_42633000	Z	42633000	57.33	50.01	49.31	67.39	4.38E-03	0.73	1.53	2
chrZ_42634000	Z	42634000	81.07	76.75	67.55	98.04	1.25E-05	0.69	1.50	2
chrZ_42635000	Z	42635000	64.55	59.29	54.35	77.36	1.73E-04	0.70	1.46	2
chrZ_42636000	Z	42636000	64.06	59.41	55.99	74.20	4.46E-03	0.75	1.43	2
chrZ_42637000	Z	42637000	87.94	82.79	77.66	100.85	1.71E-03	0.77	1.43	2
chrZ_42638000	Z	42638000	77.38	73.39	69.06	87.82	2.81E-02	0.79	1.42	2
chrZ_49068000	Z	49068000	33.32	33.10	29.34	38.32	9.41E-04	0.77	1.43	3
chrZ_49069000	Z	49069000	103.06	93.79	88.37	121.50	2.15E-04	0.73	1.41	3
chrZ_49070000	Z	49070000	73.53	64.76	61.14	89.06	1.55E-04	0.69	1.38	3
chrZ_49071000	Z	49071000	82.15	76.27	69.69	97.78	2.40E-04	0.71	1.34	3
chrZ_49072000	Z	49072000	109.91	100.09	91.25	133.31	1.73E-06	0.68	1.31	3
chrZ_49073000	Z	49073000	51.46	46.02	42.84	62.27	4.95E-04	0.69	1.27	3

### Sex differences in gene expression in the MHM Regions

To test if females up-regulate gene expression in the MHM regions, or if males down-regulate gene expression, genes that were present in the combined MHM regions were divided into those that had a significant sex difference and those that did not (see Table 1). Fourteen genes had a significant sex difference, and 22 did not (though as noted above, many still had less than the average male: female expression ratio). For the genes that displayed significant sex differences, males had an average expression of 5952 (S.D. 8804), whereas for balanced genes (no sex difference) the average male expression was 343 (S.D. 323). A similar pattern was seen for females, with unbalanced genes having an average expression of 4869 (S.D. 7227), and balanced genes having an average expression of 308 (S.D. 276). Of the two genes that displayed female biased expression (i.e. increased expression in females), the average male expression was 335 (S.D. 318), and the average female expression was 1643 (S.D. 1977). For both males and females, balanced genes had a significantly lower expression (males, t-test statistic -2.4, p = 0.03, females t-statistic = -2.4, p = 0.035). This potentially indicates that balancing occurs by decreasing male gene expression, though female gene expression is also low for these genes. Note that one issue here is that the unbalanced genes in the MHM region are still being balanced to some extent (with lower gene expression differences on average than the Z chromosome on average).

### Methylation correlations in balanced and unbalanced genes in MHM regions

To assess the role that methylation plays in sex balanced and sex unbalanced genes in the MHM regions, we tested for a difference in the number of correlations for each gene with the local methylation windows present in their respective MHM regions. In the case of unbalanced genes within the MHM regions, an average of 4.64 correlations were found per gene (S.D. 2.4). All of these correlations were positive (i.e. methylation was positively correlated with gene expression). For the unbalanced female-biased genes within the MHM region, 9 correlations were found per gene (S.D.0), with all of these having a negative correlation with methylation. In the case of the balanced genes, 0.95 correlations per gene were found (S.D. 1.9). Of these, 0.18 correlations per gene had a negative correlation and 0.77 correlations per gene had a positive correlation. Therefore sex-balanced genes (no significant sex difference in gene expression levels) show significantly fewer correlations with local MHM-region methylation (t-statistic = -4.8, p = 6.7x10^-5^), and whilst unbalanced genes all show positive correlations between methylation and gene expression, balanced genes display a mixture of positive and negative correlations. For female biased unbalanced genes, this group display the most correlations per gene, with all of these being negative. These two genes are the lncRNAs that were previously identified in the initial MHM region. These results appear to show that quantitative changes in methylation are only correlated with gene expression when no (or less) sex balancing occurs, with increasing local methylation appearing to increase, rather than decrease, gene expression. When genes are sex-balanced, local methylation appears to play less of a role in regulating gene expression.

### Methylation QTL present on the Z chromosome

Methylation QTL were assessed by performing local (cis) methylation QTL scans restricted solely to the Z chromosome. In addition, trans scans were also performed, where the QTL was located on the Z chromosome, but the target methylation window was free to be present on either the Z chromosome or the autosomes. In total, we identify 18 significant cis methylation QTL and 53 significant trans methylation QTL that are based on the Z chromosome, with a further 20 suggestive cis methylation QTL and 528 trans methylation QTL. As expected, most of the methylation QTL (n = 528) had a significant sex interaction effect. This is expected due to the large differences in Z chromosome methylation between males and females, with males possessing two methylated chromosomes (ZZ) and females only one (ZW). A full list of all methylation QTL can be found in S4 Table. In addition, 51 expression QTL (eQTL) were identified on the Z chromosome (either as a QTL or the trans-effect phenotype of a QTL), see S5 Table.

### Trans Methylation QTL Hotspots Affecting the Z chromosome

To identify trans-acting hotspots, we identified where multiple methylation QTL were associated with the same marker and had overlapping confidence intervals. Of the 619 methylation QTL on the Z chromosome, these mapped to 141 different SNP loci. Of these loci, 13 were associated with multiple methylation windows/phenotypes (10 or more methylation windows associated with each marker, respectively). These hotspots on average spanned 5.87Mb of physical distance in the genome (found by taking the shared overlapping confidence intervals and finding the minimum overlapping size), see Table 3. Of note, all bar one (n = 12) of these trans hotspots were located on the autosomes, but regulated variation in methylation on the Z chromosome. Of these 12, 3 were previously identified as regulating methylation variation on the autosomes in this intercross [4], on chromosomes 3 (at 18Mb, hotspot 4), 6 (at 7.7Mb, hotspot 6) and 7 (at 2.4 Mb, hotspot 9). One hotspot was located on the Z chromosome (at 41.7Mb, hotspot 13, with this hotspot spread over three adjacent SNPs, rs16768340, rs16782623, rs14016786, see S4 Table) and regulated variation in methylation on different windows in the Z chromosome, as well as some methylation windows on the autosomes. Thus, whilst the majority of regulation in methylation variation appears to be located on the autosomes, with these loci then regulating methylation on the Z chromosome, there is also some regulation of methylation variation by the Z chromosome itself, and even a small amount of autosomal regulation from the Z chromosome.

**Table 3:** List of trans methylation QTL hotspots. Table shows the number of methylation QTL present for each hotspot, its chromosome and base-pair position (nearest marker), and the confidence interval of each hotspot. The number of genes present within the intervals as determined by ensembl.org is also given.

id	num_qtl	marker	chr	pos	CI_low_marker	CI_high_marker	CI_low_pos	CI_high_pos	CI_size	num_genes
hotspot_1	11	Gg_rs14793763	1	13847380	Gg_rs13832402	Gg_rs15194859	12488579	14847187	2358608	50
hotspot_2	23	Gg_rs14858437	1	92741754	Gg_rs13901810	Gg_rs13910957	91200315	101131756	9931441	129
hotspot_3	11	Gg_rs15060526	2	8387159	Gg_rs14132382	Gg_rs14139143	5843745	11697701	5853956	87
hotspot_4	64	Gg_rs15282380	3	17984384	X3_16300000	Gg_rs14327472	15489694	23999342	8509648	201
hotspot_5	16	Gg_rs15416272	3	86515515	Gg_rs15403420	Gg_rs15427786	79192646	91808917	12616271	177
hotspot_6	19	X4_1267185	4	1286191	X4_1267185	Gg_rs13546113	1286191	1841819	555628	39
hotspot_7	14	Gg_rs15679503	5	22362570	snp.98.79.91070.S.2	Gg_rs15685956	17614557	25665093	8050536	184
hotspot_8	27	Gg_rs14568888	6	7742744	Gg_rs15765462	Gg_rs15777012	6568227	10633493	4065266	72
hotspot_9	11	Gg_rs15828492	7	2469584	Gg_rs15826188	Gg_rs16575534	1680380	3673817	1993437	26
hotspot_10	18	Gg_rs13609494	12	5538321	Gg_rs13621493	Gg_rs14974529	3076405	6304262	3227857	92
hotspot_11	37	rbl1871	14	15000631	Gg_rs15002638	rbl1871	13628710	15000631	1371921	52
hotspot_12	12	Gg_rs13744918	17	3944254	Gg_rs15033588	Gg_rs13744523	2473253	5465495	2992242	76
hotspot_13	39	GG_rs16782623	Z	41699011	Gg_rs16768340	Gg_rs16114279	37374725	52128174	14753449	176

**Fig 3:**
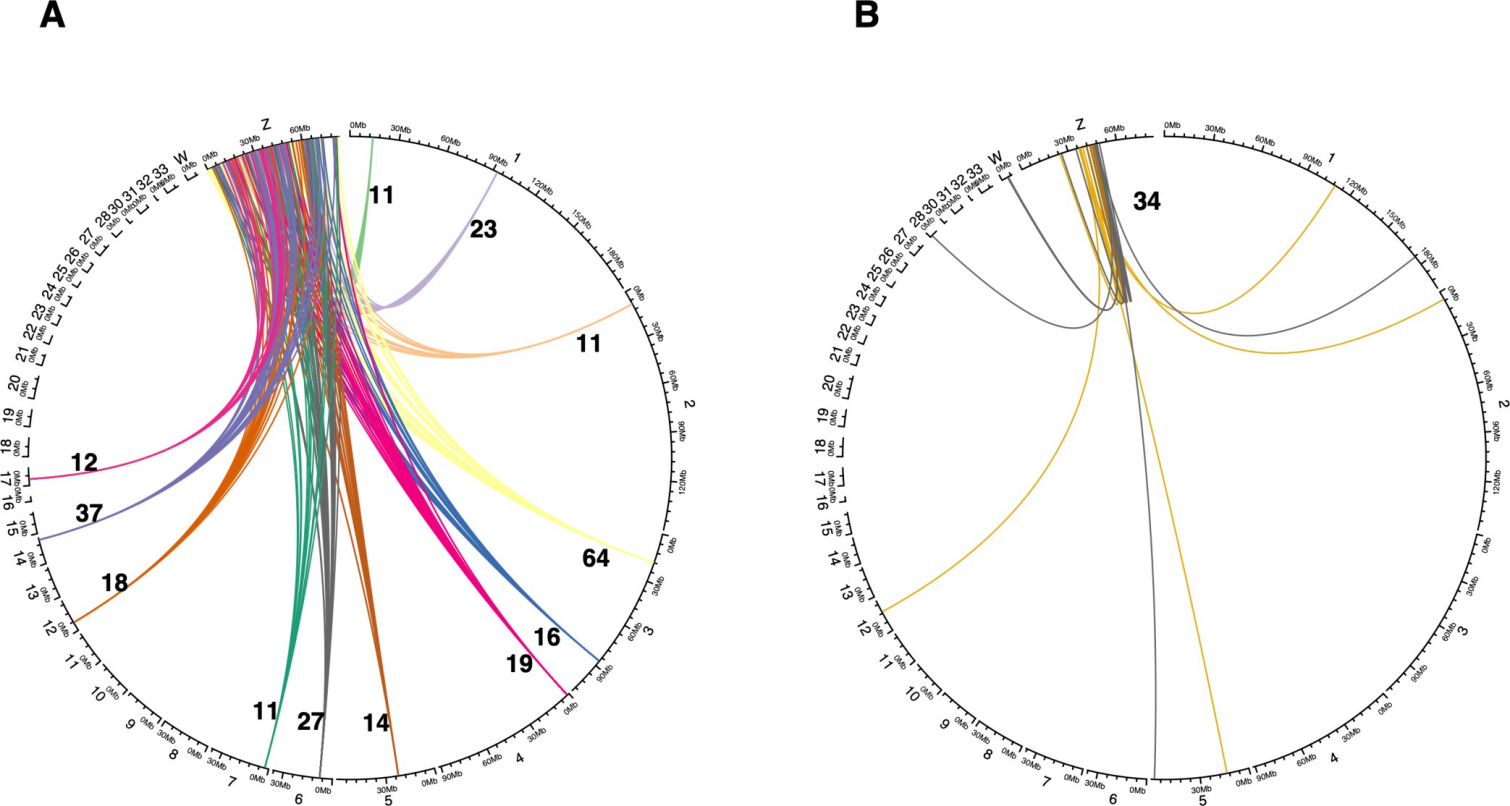
Circle plot showing the location of trans methylation QTL hotspots that affect DNA methylation variation on the Z chromosome. (A) The 12 autosomal hotspots affecting Z DNA methylation, and (B) the single Z chromosome hotspot affecting Z and autosomal methylation.

The genes present within these hotspots were further checked for potential enrichment via gene ontology analysis, using DAVID 6.8 (https://david.ncifcrf.gov/). In total 3 hotspots showed enrichment using the DAVID 6.8 database: the hotspot (ID#2) at chr1@91.7MB contained genes enriched for immunoglobulin-fold/domain, the hotspot (ID#5 in Table 3) on chr3@86.5Mb had genes enriched for the activity of glutathione and metabolism of cytochrome P450, and the hotspot (ID#6 in Table 3) on chr4@1.3Mb contained genes enriched for activity with rhodopsin, see S6 Table. The hotspots and their distribution across the genome are illustrated in Fig 3. Gene enrichment analysis was also performed for the target genomic regions in the vicinity (±10kb) of each methylation window associated with a methylation QTL hotspot. Some enrichment was found for hotspot ID#4 (located on chromosome 3 at 17.98Mb), however, this result was non-significant (Bonferroni *p*-value > 0.05).

### Role of cis and trans methQTL in sex balancing on the Z chromosome

To test the role that cis and trans regulation plays in sex-balancing gene expression on the Z chromosome, we selected all the cis-acting methQTL that were present on the Z chromosome, as well as any trans acting loci that affected methylation on the Z chromosome. These QTL were then divided into those with a significant sex-interaction, and those without one. In this case, a sex interaction indicates that the allelic effect of the QTL is different depending on the sex of the recipient, i.e. an interaction between QTL and sex is identified. Note that this is distinct from the fixed factor effect of sex (i.e. when one sex always has a uniformly higher or lower expression relative to the other). Thirty-eight cis methQTL were present on the Z chromosome, and none of these had a significant sex-interaction. In stark contrast, 540 trans methQTL affected Z chromosome methylation, and 528 of these displayed a significant sex interaction, with only 22 not displaying such an interaction (chi-squared test, p<0.0001). This ratio is similar even if only significant and not suggestive loci are considered. In this case, 45 trans methQTL showed a sex interaction effect, and only 3 did not.

### Sex differences in eQTL gene expression and the Z chromosome

To test how sex balanced and sex unbalanced genes are differently regulated over the whole Z chromosome, all of the 51 eQTL that were either present on the Z chromosome or affected gene expression on the Z chromosome (but were trans in effect) were assessed. The trans effects that were located on the autosomes but affected gene expression on the sex chromosome (n = 43) all had a significant sex interaction. On average, gene expression for these genes in males was 3276 (S.D. 6521), and 2923 (S.D. 6082) in females. In stark contrast, all the cis-acting eQTL that were present on the Z chromosome had no significant sex interaction effect. Similarly, all the trans-acting eQTL that were based on the Z chromosome, but affected autosomal gene expression, also had no significant sex interaction. The average gene expression values of the genes with no significant sex interaction was 381 (S.D. 549) for males, and 243 (S.D. 246) for females.

### Causality analysis between Methylation and gene expression on the Z chromosome

In total 360 overlaps were found between eQTL and methylation QTL. These were methylation and expression QTL where either the QTL or methylation phenotype were located on the Z chromosome. The overlapping phenotypes (gene expression and methylation) were tested for association using a linear model. Of these, 15 overlaps were significant after applying an FDR-based multiple testing corrections. Eleven of the overlaps were significant (p-value < 0.05, FDR corrected) using all individuals, while 3 were significant (p-value < 0.05, FDR corrected) using only females, and 1 was significant (p-value < 0.05, FDR corrected) using only males, see Table 4. These overlaps contained 5 unique probesets belonging to 2 unique genes and 3 ESTs. The gene LINGO1 (*ENSGALG00000002708*; chr10:3212741–3290778) is an immunoglobulin domain protein [37]. Immunoglobulin activity was also found in the methylation QTL hotspot on chromosome 3. Additionally, the 15 overlaps were tested with NEO, a network edge orientated method which uses the underlying QTL genotype as anchors for the network [36], to assess the orientation of the observed correlation. In this case, this means testing whether the causality relationship indicates that methylation is driving gene expression, or the reverse (or if the two are unrelated). Four of the overlaps had a LEO.NB.OCA-score > 0.3. Both eQTL and mQTL originated from the same genotype (imputed marker position) and thus are treated as a single-marker orientation where a LEO.NB.OCA-score > 1.0 is significant. Hence, our results indicate that the EST X603598164F1 (gene id: *ENSGALG00000050497*, *chrZ*:*44706094–44707218*) influences the methylation levels in the region of chrZ:45163000–45165000, see Table 4. This gene has been retired on the GalGal6 genome, with no known function. In addition, one further EST (X603865974) was suggestive (LEO.NB.OCA >0.8), with methylation appearing to drive gene expression in this case. However, as the model p-value was significant, this means that other models (gene expression driving methylation) cannot be ruled out.

**Table 4:** NEO causality of gene regulation of methylation. The probeset and the methylation window being tested, along with their confidence interval is presented. In addition, the genotype p-value, the sex p-value (also broken down into male and female), as well as the actual causality statistics (leo.nb.oca and cpa and the model p-value) are all shown.

probeset	eqtlCI	methbin	mqtlCI	pvalue	sexpvalue	male_pvalue	female_pvalue	ensembl_id	gene_name	gene_pos	neo_edge	LEO.NB.OCA	LEO.NB.CPA	model_pvalue
X603602693F1	chrX:317.15–360.02	chrZ_8007000	chrX:66.27–353.15	3.5E-01	6.3E-01	8.8E-01	3.1E-02	#N/A	#N/A	chrZ:39822637–39823353	meth -> genexp	0.44	0.44	0.25
X603602693F1	chrX:317.15–360.02	chrZ_45165000	chrX:335.14–348.56	8.6E-02	3.2E-01	7.2E-01	3.8E-02	#N/A	#N/A	chrZ:39822637–39823353	meth -> genexp	0.03	0.03	0.06
X603602693F1	chrX:317.15–360.02	chrZ_45166000	chrX:335.14–348.56	3.5E-01	1.4E-01	9.2E-01	4.2E-03	#N/A	#N/A	chrZ:39822637–39823353	n.s			
X603598164F1	chrX:317.15–408.11	chrZ_43720000	chrX:317.15–353.15	1.2E-02	1.3E-09	8.1E-02	8.6E-01	ENSGALG00000050497	#N/A	chrZ:44706094–44707218	n.s			
X603598164F1	chrX:317.15–408.11	chrZ_43860000	chrX:335.14–353.15	3.9E-03	1.3E-09	1.7E-02	9.6E-01	ENSGALG00000050497	#N/A	chrZ:44706094–44707218	n.s			
X603598164F1	chrX:317.15–408.11	chrZ_43861000	chrX:335.14–348.56	2.5E-03	4.8E-10	3.5E-02	7.4E-01	ENSGALG00000050497	#N/A	chrZ:44706094–44707218	n.s			
X603598164F1	chrX:317.15–408.11	chrZ_43862000	chrX:0–353.15	1.3E-02	1.3E-09	8.3E-02	9.8E-01	ENSGALG00000050497	#N/A	chrZ:44706094–44707218	n.s			
X603598164F1	chrX:317.15–408.11	chrZ_45164000	chrX:335.14–348.56	1.0E-03	1.9E-06	1.7E-02	7.3E-01	ENSGALG00000050497	#N/A	chrZ:44706094–44707218	genexp -> meth	1.60	1.60	0.21
X603598164F1	chrX:317.15–408.11	chrZ_45165000	chrX:335.14–348.56	6.2E-04	2.2E-05	1.7E-02	7.4E-01	ENSGALG00000050497	#N/A	chrZ:44706094–44707218	genexp -> meth	2.13	2.13	0.05
X603598164F1	chrX:317.15–408.11	chrZ_45166000	chrX:335.14–348.56	3.3E-02	1.6E-06	2.1E-01	5.8E-01	ENSGALG00000050497	#N/A	chrZ:44706094–44707218	n.s			
X603598164F1	chrX:317.15–408.11	chrZ_46826000	chrX:0–408.11	3.7E-02	1.4E-08	2.1E-01	7.0E-01	ENSGALG00000050497	#N/A	chrZ:44706094–44707218	n.s			
X603598164F1	chrX:317.15–408.11	chrZ_47304000	chrX:348.56–360.02	3.3E-02	4.5E-05	3.7E-01	2.2E-02	ENSGALG00000050497	#N/A	chrZ:44706094–44707218	n.s			
X603865974F1	chrX:317.15–408.11	chrZ_47304000	chrX:348.56–360.02	5.4E-02	7.5E-02	4.6E-02	7.3E-01	#N/A	#N/A	chrZ:49932148–49932712	meth -> genexp	0.94	0.94	0.04
X603862692F1	chrX:317.15–335.14	chrZ_29446000	chrX:0–335.14	2.5E-03	9.9E-01	8.7E-01	4.2E-03	#N/A	#N/A	chr3:48355661–48356479	n.s			
X603602881F1	chrX:317.15–335.14	chrZ_29446000	chrX:0–335.14	2.5E-03	7.5E-01	9.6E-01	4.2E-03	ENSGALG00000002708	LINGO1	chr10:3212741–3290778	meth -> genexp	0.05	0.05	0.96

## Discussion

Using this wild x domestic paradigm to analyse DNA methylation and its regulation on the Z chromosome in the chicken, we firstly identify over 600 methylation QTL that affect methylation on the Z chromosome. Of these, the majority of trans effect loci are located on the autosomes but affecting the Z chromosome. There were also examples of the reverse, with trans methylation QTL deriving from the Z chromosome but affecting DNA methylation on the autosomes. Furthermore, these trans methylation QTL were concentrated into a small number of hotspots located on the autosomes (n = 12), with one hotspot also present on the Z chromosome itself, associated with methylation on the Z chromosome and the autosomes, respectively. A total of five genes on the Z chromosome were also candidates for causality between gene expression and methylation, with two passing the network-edge-based threshold for significance. Of these, one appears to be a retired gene, whilst the other is an EST of no known function, with the former indicating gene expression affects methylation, whilst the latter indicates that methylation was modifying gene expression.

We also assessed the role of methylation and gene expression in the regulation of sex-balancing in both the new and old MHM regions. Sex-balanced genes had decreased expression in the MHM region, relative to the unbalanced/ less balanced genes that were present. This suggests that for these genes, male expression is reduced, with a similar effect to mammalian XY systems. The expression of the balanced genes was less than half the expression of the unbalanced genes, implying that these genes are even more tightly regulated in both sexes. The relationship with DNA methylation in sex balanced and sex unbalanced (or less balanced) genes was also assessed, with a strong difference found with balanced genes displaying far fewer correlations with local methylation than unbalanced genes. In addition, unbalanced genes only displayed positive correlations with local methylation. These indicate that strongly sex-balanced genes are not affected by local methylation, but more weakly balanced or unbalanced genes are more likely to be regulated by local methylation (or at least correlated with it).

The presence of sex interactions in both the methylation and expression QTL that were identified can also provide evidence for the regulation of sex balancing on the Z chromosome. With regard to methylation QTL, the local (cis) methQTL were all strongly balanced, whereas the non-Z chromosome based trans methQTL were all unbalanced in that they displayed strong sex interactions with each QTL. This appears to show that locally-regulated methylation is very stable between the sexes–even when there may be differences in total methylation between the sexes at these loci, these are not interacting with allelic effects and regulation. In contrast, trans-effect loci seem distinct from the sex-balancing effects. Almost all of these are located on the autosomes and show modified Z chromosome methylation levels, implying that they are not part of the sex-balancing mechanisms that exist on the Z chromosome, and could even be a way of introducing more variation between the sexes in terms of methylation regulation on the Z chromosome.

Sex differences in expression QTL on the Z chromosome displayed a similar pattern as methylation QTL, with cis-acting loci on the Z displaying sex balance (or more accurately a lack of any sex interaction with the eQTL), whereas trans-acting loci displayed a large degree of sex-interaction when they originated from the autosomes. Also similar to the genes on the MHM region, both male and female expression values for the sex balanced genes were significantly lower than the unbalanced genes. These results imply that when the cis and trans effects originate from the Z chromosome, whatever factor is balancing these genes remains, and that potentially the genes affected are not related to sex-biased expression traits. In contrast, when the QTL are present on the autosomes, whatever factor that balances expression between the sexes is bypassed, with these genes more likely to be related to sex-biased trait expression due to the large differences that can be induced between the sexes by the alleles in question.

Intra-locus sexual conflict arises from differential selection pressures experienced by males and females, when they share a common genetic basis [38]. This can be resolved when a locus has different effect between the sexes, with this being considered as a separate genetic architecture between the sexes [39], with several examples of phenotypic traits having such a distinct genetic architecture between the sexes [40, 41, 42]. Regulatory evolution is a rapid and efficient solution to intra locus sexual conflict [43], where the regulatory elements of a gene evolve to act in a sex-specific manner. Sex-interacting expression QTL and methylation QTL represent exactly this type of differential genetic architecture between the sexes. The results shown here would therefore suggest that it is possible to induce such separate genetic architecture for genes on the Z chromosome, but only when the QTL are located on the autosomes. When regulatory loci are present on the Z chromosome itself, these do not exhibit this differential architecture, and appear instead to always give equal effects between the sexes. Therefore, the autosomal-derived trans effects appear to drive sex differences (and thus reduce sexual conflict), whereas Z chromosome-based cis and trans effects appear to be unable to escape the dosage compensation and/ or other sex balancing effects that exist on the Z chromosome.

The nature of the intercross (a wild bird intercrossed with domestics) allows us to identify consistent differences in methylation that exist between wild and domestic chickens and the regions that associate with and potentially regulate them. With regards to the methylation QTL hotspots identified, it is noteworthy that these are almost all based on the autosomes, with only one situated on the Z chromosome itself. Therefore, the regulation of domestication-based phenotypes with loci present on the Z chromosome appears to generally be autosomally regulated, although the reverse (where autosomal gene expression is regulated by the sex chromosomes) also occurs. Interestingly, three of the hotspots previously identified as regulating DNA methylation in domestication (primarily via reducing DNA methylation in domestic birds) also appear to regulate DNA methylation on the Z chromosome [4].

As well as the regulation of variation in methylation, we also identified additional Male Hyper-Methylated regions present on the Z chromosome. Unlike the initial MHM region found [24], which identified that the lncRNAs present were completely switched off in males, the regions we identify appear to instead decrease male gene expression, though rather than reduce it entirely, it is instead down-regulated to levels more closely found in females (i.e. reduced male gene expression, relative to female gene expression). This is despite the regions having a similar pattern of sex-differentiated methylation as is seen in the original MHM region. Further, the strength of the methylation differences between sexes was greater in the new regions we identified when compared to the region at 74Mb (although we also identify the 74Mb MHM region as well). These genes thus appear to be linked to sex-based differences between males and females. No methylation QTL overlap these regions, implying that these regions are not responsible for regulating variation in methylation, which would then fit with these regions instead regulating more basal sex-differences rather than between-population variation. This idea is reinforced when considering the functions of the genes in these regions.

Of the 18 known genes that are present within the MHM regions, their functions can be broadly divided into learning/ behaviour, bone allocation, development, reproduction, growth/ metabolism and methyl transferase activities. These tie-in well with the known sex-differences that exist in the chicken. Starting with behaviour, strong behavioural differences exist between males and female chickens [10, 44, 45, 46]. In particular, females have decreased anxiety-related behaviour, though this may be test-dependent [30,47,48,49,50]. Of the genes present in the MHM, four are related to behaviour or neurogenesis. The gene *SLC1A1* has been shown to play a role in obsessive compulsive disorder and sterotype behaviour [51,52], as well as schizophrenia susceptibility [53,54]. Anxiety behaviour in chickens has previously been shown to be related to schizophrenia, depression and other mood-based disorders in humans, even sharing some of the same susceptibility loci [30,47]. Furthermore, the OCD effects arising from *SLC1A1* are stronger in males, so sex-differences in the gene effects have already been demonstrated [55,56]. ZDHHC2I is a major palmitoyl acyltransferase, with decreasing expression leading to increased depression-like behaviours [57]. *Homer1* also has functions relating to learning and memory [58], and also causes susceptibility to Alzheimers [59]. In the case of the latter, these effects are strongly sex-dependent, only occurring in women.

Continuing with bone allocation, female chickens have a complex bone allocation, whereby during egg production the hard outer cortical bone is first mobilised into soft, spongy medullary bone in the centre of the femur, before then being transferred to create the egg shell (one of the major limiting factors in egg production) [60,61]. Therefore male and female chickens differ markedly in their bone metabolism–males possess almost no medullary bone, whilst female medullary bone deposition is strongly associated with reproductive output [29,33,62]. Of the genes in the MHM, *Homer1* has numerous beneficial effects in osteoblasts including beta-catenin stabilization [63]. *FST* (foillistatin) is also a powerful regulator of bone metabolism [64]. *CER1* has also been found to regulate bone mineral density and be associated with fracture risk. Of note, these effects are found to be strongest in post-menopausal women [65,66]. *TLE4* is a critical mediator of osteoblasts and *runx2-*dependent bone development in the mouse [67]. Finally, *NANS* affects skeletal development in zebrafish knock-outs [68]. Continuing with reproduction-related genes, the gene *FST* plays a critical role in mouse uterine receptivity and decidualization [69], whilst the gene *JMY* mediates spermatogenesis in mice [70] as well as asymmetric division and cytokinesis in mouse oocytes [71]. Finally, *CLTA4* is involved in the maintenance of chronic inflammation in endometriosis and infertility [72].

The final category of genes present in the MHM regions affected growth and metabolism, whilst two methyltransferase genes were also present. Large differences in growth and bodyweight exist in the chicken, with males often twice the bodyweight of females. The gene *FST*, as well as affecting reproduction-related phenotypes, also leads to increased muscle weight in mice when over-expressed [73]. *DMGDH* affects body growth through insulin-like growth factor [74], whilst also affecting selenium status in pregnant women [75]. *ABHD3* regulates adipogenic differentiation and lipid metabolism [76]. Finally, *BHMT* is a methyltransferase, as is *DMGDH*.

In the current study, we have restricted the investigation to a single tissue, albeit repeated in 124 individuals. As such, we are confident that these MHM regions and methylation QTL are present in this tissue type. However, the ubiquity of these methylated regions in other tissues must be verified. This opens up the possibility that multiple further MHM regions also exist, but are only present in specific tissue types. This could allow fine-scale regulation of sex differences in a tissue-specific manner. We also assess female-specific hyper-methylated regions, but these were found to be very sparse and had very few genes present, suggesting these are of less importance. In summary, by using a large number of replicates that are assessed for all methylated loci on the Z chromosome, we identify both novel MHM regions in an intra-specific/ inter-population framework, as well as the role that domestication plays in the regulation of the Z chromosome and the genes on it.

The use of eQTL and methQTL studies can help to understand sex differences and sex-balancing as related to the Z chromosome, as well as sexual conflict resolution. Our results indicate that Z chromosome sex-balancing regulation appears to decrease male gene expression for the most tightly regulated genes, whilst the role of local methylation seems to be stronger for more weakly balanced genes or unbalanced genes. Given that methylation QTL and expression QTL studies deal explicitly with within-population quantitative variation, they represent an excellent resource with which to understand the more fine-scale sex-balancing that is occurring, as opposed to the very severe dosage compensation and sex-balancing effects seen in the classical MHM regions.

## Conclusions

Sex balancing is not only vital to regulate basic gene expression between the sexes on the sex chromosomes, but also as a mechanism to resolve intra-locus sexual conflict. Our results indicate that the MHM region contains several examples of quantitative sex-balancing effects occurring in the genes present there. This implies that this region is not purely limited to a means of removing expression of key lncRNAs in males. We also find that sex-balancing is not limited to just the MHM region, but also occurs throughout the Z chromosome via trans-effect autosomally-based QTL. These results agree with the study by Catalan et al [77] that found methylation was spread throughout the Z chromosome, and that questioned the role of the MHM in dosage compensation. Whilst our results don’t address dosage compensation, they do indicate that sex balancing also operates on a gene-by-gene basis, at least to some extent, and that the autosomes may have an important role in such regulation. In contrast, Z chromosome-based modifier loci appear to be limited in their ability to modify Z chromosome gene expression independently between the sexes.

## Supporting information

S1 Fig All of the Novel MHM regions present on the Z chromosome and their effects on gene expression. Each pane consists of the following: i) The male:female methylation ratio for the 1kb methylation windows that make up the MHM region (each black dot represents the ratio at one methylation window). The red hashed line at the base indicates the average male:female methylation ratio (~1.7). ii) Male:female gene expression ratio is indicated by the blue dots, one for each gene in the region, with the ratio shown on the left-side y-axis, and the blue hashed line indicating the average male:female gene expression ratio on the Z chromosome (~1.2). iii) The number of correlations between each gene and the 1kb methylation windows that make up each MHM. The direction of the correlation (positive or negative) is indicated by the bar being above the line (positive, coloured turquoise) or below the line (negative, coloured purple). The number of correlations is indicated on each bar, whilst each gene name is given on the x-axis.
(PDF)

S1 Table MHMa and MHMb regions. The position, average and median methylation per window, and the average methylation in males and females per window are all given, as well as the significance of the sex-difference and the average male:female fold ratio.
(XLSX)

S2 Table The previously identified MHM region at 73Mb. The position, average and median methylation per window, and the average methylation in males and females per window are all given, as well as the p-value and significance of the sex-difference and the average male:female fold ratio. Note for the significance of the sex difference, these are classified as non-significant, significant (including a multiple testing correction), and significant at the same level as the original MHM region.
(XLSX)

S3 Table All female hyper-methylated regions. The three FHM blocks (continuous regions) are highlighted in orange and indicated with their block ID in a separate column. The position, average and median methylation per window, and the average methylation in males and females per window are all given, as well as the significance of the sex-difference and the average male:female fold ratio.
(XLSX)

S4 Table List of local (cis) and trans methylation QTL present on the Z chromosome. The phenotype of each methylation QTL (methylation window), nearest marker to the methylation QTL, LOD score, confidence interval, and nearest marker to each confidence are given, as well as whether the QTL is cis or trans in effect, are all given.
(XLSX)

S5 Table Expression QTL (eQTL) present on the Z chromosome. Closest marker, LOD score, confidence interval, presence or absence of sex interaction, and nearest marker to the confidence interval are all presented.
(XLSX)

S6 Table GO enrichments from methylation QTL hotspots. Category, GO term, p-value (absolute and also FDR controlled), genes involved and fold enrichment are all given.
(XLSX)
