# Supplementary material for: The regulation of methylation on the Z chromosome and the identification of multiple novel Male Hyper-Methylated regions in the chicken
Source: PLoS Genet. 2024 Mar 8;20(3):e1010719. doi: 10.1371/journal.pgen.1010719 (PMC10954189; doi:10.1371/journal.pgen.1010719)
Supplement: S1 Fig — Each pane consists of the following: i) The male:female methylation ratio for the 1kb methylation windows that make up the MHM region (each black dot represents the ratio at one methylation window). The red hashed line at the base indicates the average male:female methylation ratio (~1.7). ii) Male:female gene expression ratio is indicated by the blue dots, one for each gene in the region, with the ratio shown on the left-side y-axis, and the blue hashed line indicating the average male:female gene expression ratio on the Z chromosome (~1.2). iii) The number of correlations between each gene and the 1kb methylation windows that make up each MHM. The direction of the correlation (positive or negative) is indicated by the bar being above the line (positive, coloured turquoise) or below the line (negative, coloured purple). The number of correlations is indicated on each bar, whilst each gene name is given on the x-axis. (PDF) [file pgen.1010719.s001.pdf]

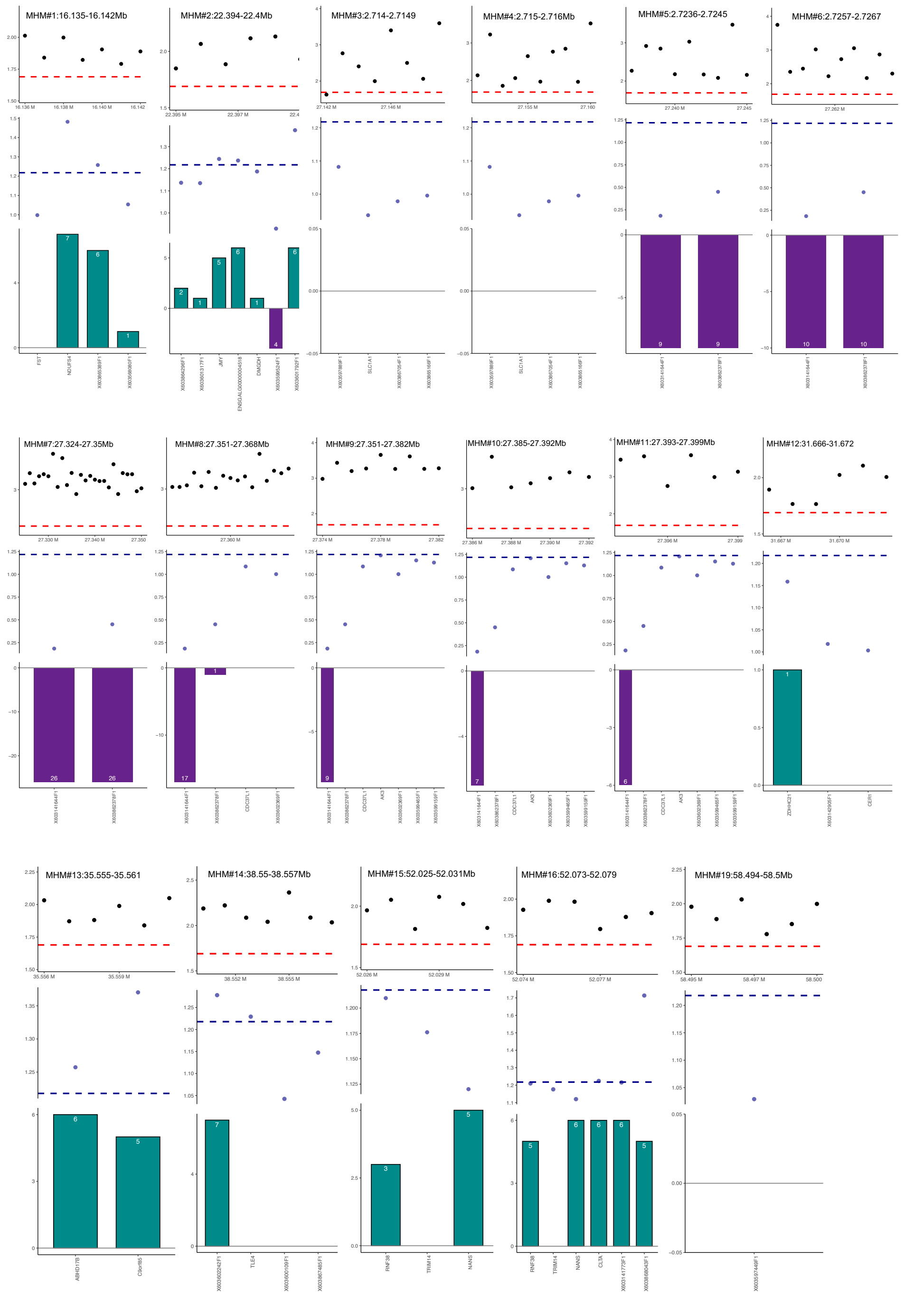
MHM#1:16.135-16.142Mb
FST
NDUFS4
X603865389F1
X603568080F1
MHM#2:22.394-22.4Mb
X603864296F1
X603601317F1
JMY
ENSGALG00000004518
DMGDH
X603599524F1
X603601792F1
MHM#3:2.714-2.7149
X603597889F1
SLC1A1
X603867054F1
X603865166F1
MHM#4:2.715-2.716Mb
X603597889F1
SLC1A1
X603867054F1
X603865166F1
MHM#5:2.7236-2.7245
X603141644F1
X603862378F1
MHM#6:2.7257-2.7267
X603141644F1
X603862378F1
MHM#7:27.324-27.35Mb
X603141644F1
X603862378F1
MHM#8:27.351-27.368Mb
X603141644F1
X603862378F1
CDC37L1
X603602369F1
MHM#9:27.351-27.382Mb
X603141644F1
X603862378F1
CDC37L1
AK3
X603602369F1
X603599465F1
X603599159F1
MHM#10:27.385-27.392Mb
X603141644F1
X603862378F1
CDC37L1
AK3
X603602369F1
X603599465F1
X603599159F1
MHM#11:27.393-27.399Mb
X603141644F1
X603862378F1
CDC37L1
AK3
X603602369F1
X603599465F1
X603599159F1
MHM#12:31.666-31.672
ZDHHC21
X603142905F1
CER1
MHM#13:35.555-35.561
ABHD17B
C9orf85
MHM#14:38.55-38.557Mb
X603602242F1
TLE4
X603600109F1
X603687485F1
MHM#15:52.025-52.031Mb
RNF38
TRIM14
NANS
MHM#16:52.073-52.079
RNF38
TRIM14
NANS
CLTA
X603141773F1
X603868043F1
MHM#19:58.494-58.5Mb
X603597449F1
